# Melatonin and tannic acid supplementation *in vitro* improve fertilization and embryonic development in pigs

**DOI:** 10.21451/1984-3143-AR2016-937

**Published:** 2018-08-16

**Authors:** Rachel L. Lane, Brian D. Whitaker

**Affiliations:** 1 Department of Animal and Pre-veterinary Studies, University of Findlay, Findlay OH, 45840, USA.

**Keywords:** IVF, melatonin, oocyte maturation, oxidative stress, tannic acid

## Abstract

The objective of this study was to determine the effects of melatonin supplementation during maturation and tannic acid supplementation during IVF on fertilization kinetics and early embryonic development. Experiment 1 determined the optimum concentration of melatonin supplemented to the oocytes for subsequent embryonic development. Oocytes (n = 400) were supplemented at 22 h of maturation with 0, 75, 100, or 150 nm melatonin and then subjected to IVF and embryo culture. After IVF, a portion of the embryos were evaluated for penetration, polyspermy, and male pronuclear (MPN) formation rates. Embryos were evaluated 48 h after IVF for cleavage and 144 h for blastocyst formation. There were no significant differences between treatment groups with respect to penetration and polyspermy. Supplementation of 150 nm melatonin produced a significantly greater (P < 0.05) percent of embryos with MPN compared to those supplemented with 75 nm or 100 nm. Supplementation of 150 nm melatonin produced significantly less (P < 0.05) embryos cleaved by 48 h after IVF while 75 nm melatonin supplementation had a significantly higher (P < 0.05) percentage of blastocyst formation by 144 h after IVF. Based on the optimal concentration of melatonin observed in experiment 1, experiment 2 determined the effects of supplementing 75 nm melatonin to the maturation media and 5.0 μg/ml tannic acid supplementation during IVF on oxidative stress, fertilization kinetics, and embryonic development. Oocytes (n = 720) were supplemented at 22 h of maturation with or without 75 nm melatonin and then fertilized with frozen-thawed sperm supplemented with or without 5 μg/ml tannic acid. Reactive oxygen species levels were measured in matured oocytes using 2’,7’-dichlorodihydrofluorescein diacetate. Oocytes supplemented with 75 nm melatonin had significantly less (P < 0.05) reactive oxygen species generation and oocytes fertilized with sperm incubated with tannic acid had a significantly less (P < 0.05) incidence of polyspermic penetration compared to no supplementation. All treatment groups had significantly greater (P < 0.05) incidence of male pronuclear formation compared to oocytes not supplemented with melatonin and fertilized without tannic acid. Oocytes that were supplemented with melatonin and fertilized with sperm incubated with tannic acid had a significantly higher (P < 0.05) percentage of blastocyst formation by 144 h post-IVF compared all other treatment groups. Results indicate that supplementation of 75 nm melatonin during oocyte maturation and 5 μg/ml tannic acid during IVF leads to a decrease in oxidative stress, increase in IVF success and subsequent embryo development in pigs.

## Introduction

High levels of reactive oxygen species (ROS) in and around maturing oocytes lead to oxidative stress, which hinders otherwise successful fertilization. High frequency of polyspermic penetration also presents a major obstacle to the production of *in vitro* derived pig embryos. Research focusing on how oocytes alleviate oxidative stress has shown the negative impact of ROS on embryonic development ( Abeydeera *et al*., 1998 ; Whitaker and Knight, 2004 ). Melatonin is a free-radical scavenger which can cross cell membranes and the blood-brain barrier ( Reiter *et al*., 2010 ; Pohanka, 2011 ). Additionally, melatonin interacts with other antioxidants to improve the overall effectiveness of each antioxidant ( Arnao and Hernández-Ruiz, 2006 ). Supplementation of melatonin has shown to lower ROS levels during oocyte maturation in mice ( Salehi *et al*., 2014 ), bovine ( Cebrian-Serrano *et al*., 2013 ) and humans ( Wei *et al*., 2013 ). In pigs, melatonin his been shown to support oocyte maturation and embryo culture (Do *et al.*, 1015) and to protect the oocyte against ROS ( Li *et al*., 2015 ).

Polyspermic penetration in porcine oocytes is still a major challenge for researchers and remains around 30% in most laboratories (Abedyeera *et al.*, 1998; Fan and Sun, 2004 ). Supplementation of tannic acid during *in vitro* fertilization (IVF) reduces polyspermy in porcine oocytes by inhibiting hyaluronidase activity, thus reducing polyspermic penetration ( Tatemoto *et al*., 2006 ).

To our knowledge, research has not been published focusing on the supplementation of both melatonin to porcine oocytes during maturation and tannic acid to frozen-thawed boar spermatozoa during IVF. Therefore, the objective of this study was to determine the effects of melatonin supplementation to the pig oocyte maturation media and 5 μg/ml tannic acid to the IVF media on oxidative stress, IVF kinetics and embryonic development. The production of ROS was measured in matured oocytes to determine oxidative stress levels. Oocyte fertilization, polyspermy, and male pronuclear (MPN) formation were observed to determine the IVF kinetics. Embryos were evaluated for cleavage and blastocyst formation.

## Materials and Methods

### Media

Unless otherwise stated, all chemicals were purchased from Sigma-Aldrich Co. (St. Louis, MO, USA). The oocyte maturation media was Medium 199 (M199) with Earle’s salts (Fisher Scientific, Pittsburgh, PA, USA) supplemented with 5 µg/ml follicle stimulating hormone (FSH), 1 µl/ml insulin, 50 ng/ml gentamicin sulfate, 10 ng/ml epidermal growth factor, and 10% fetal calf serum (v/v; FCS). The IVF medium used was a modified Tris-buffered media formulated by Abeydeera and Day (1997) . The embryo culture medium used was North Carolina State University (NCSU) 23 medium ( Petters and Wells, 1993 ) containing 0.4% (w/v) bovine serum albumin (BSA). All media were filtered through a 0.22 μm pore MCE membrane (Fisher Scientific, Pittsburgh PA, USA) syringe filter. All incubations were carried out under mineral oil at 38.5°C in an atmosphere of 5% CO2 unless otherwise indicated.

### Maturation of oocytes

Oocytes were aspirated from mature follicles (3-6 mm diameter) obtained from adult crossbred sows (at least 18 months of age) at a local abattoir. The average elapsed time between ovary collection and follicular aspiration was 3 h. Oocytes surrounded by a compact cumulus cell mass and uniform ooplasm were washed three times and placed (45-55 oocytes/well) into 500 μl of maturation medium. After 20-24 h from initial placement in media, oocytes were washed three times in maturation media and placed (45-55 oocytes/well) into 500 μl of maturation media without FSH and FCS for an additional 18-26 h. After incubation, cumulus cells were removed from the oocytes by repeat pipetting in M199 containing 0.1% hyaluronidase (w/v). Only oocytes observed with uniform granulated cytoplasm and an extruded polar body were washed in IVF medium and used as described below.

### Measuring ROS production

Levels of ROS in matured oocytes were measured by incubating the oocytes in 0.3% (w/v) BSA in PBS with 5 μm 2’,7’-dichlorodihydrofluorescein diacetate (DCHF-DA) for 30 min. Oocytes were then examined using fluorescent microscopy (excitation maximum wavelength = 490 nm and emission maximum wavelength 520 = nm), their images were recorded digitally and the fluorescence brightness at the equatorial section of each oocyte stained by DCHF-DA was calculated using computer software (Nikon NIS Elements; Nikon Instruments Inc., Melville, NY, USA). The data were presented as the percentage of fluorescent intensity present in the oocytes matured without supplementation.

### Spermatozoa preparation

Three frozen semen pellets, one from each of three different boars (International Boar Semen, Eldora, IA, USA) were thawed in IVF media and centrifuged at 36.3 x *g* for 5 min. The semen was then washed twice at 553 x *g* for 5 min. After washing, the spermatozoa pellet was re-suspended in IVF media at a concentration of 2.0 x 10^5^ sperm cells/ml and incubated for 1 h before 50 μl was added to each group of oocytes in 50 μl droplets of IVF medium. Immediately prior to IVF, sperm were analyzed for forward progressive motility using a phase-contrast microscope at 400X magnification and their viability/membrane integrity was assessed by staining with 0.6% Eosin red (w/v) and 5.0% Aniline blue (w/v) dye to ensure a quality sample of sperm being used for IVF. The frozen-thawed sperm samples used averaged 64.8 ± 5.0% forward progressive motility and 70.9 ± 5.0% live.

After 4-6 h of IVF, the putative zygotes were washed three times and placed (50 zygotes per well) into 500 μl of embryo culture medium and incubated. Embryos were evaluated for cleavage and blastocyst formation under a stereomicroscope at 48 and 144 h after IVF, respectively.

### Examination of IVF characteristics

Approximately 12 h after IVF, oocytes were mounted and fixed with 25% acetic acid in ethanol (v/v) at room temperature. After 48 h of fixation, oocytes were stained with 1% orcein (w/v) in 45% acetic acid (v/v) and examined using a phase-contrast microscope at 400X magnification. Oocytes were characterized by visualization of penetration, MPN formation, and polyspermic penetration. Oocytes were considered penetrated when they had one or more swollen sperm head(s) or MPN and their corresponding sperm tails.

### Experimental design

#### Experiment 1: determination of optimum concentration of melatonin for embryo production

Oocytes were matured in maturation media for 20-24 h and then in fresh maturation media without FCS and hormones for an additional 18-26 h. During the second stage of maturation, the fresh media was supplemented with 0, 75, 100 or 150 nm melatonin. After IVF, the number of oocytes penetrated was recorded (n = 200; 50 oocytes/treatment group). Of those penetrated, the number of polyspermic oocytes, and the number of oocytes penetrated with a MPN were determined. During embryonic development (n = 400; 100 oocytes per treatment group), the number of embryos cleaved at 48 h after IVF and blastocysts at 144 h after IVF were observed and recorded. A total of 600 matured oocytes (150 oocytes/treatment group) over two replicates were used in this experiment.

#### Experiment 2: effects of melatonin and tannic acid supplementation on IVF and embryonic development

Experiment two supplemented 75 nm melatonin to the maturation media, since it elicited the lowest rate of polyspermic penetration and highest penetration rate, MPN formation and cleavage and blastocyst formation rate. The effects of supplementing 75 nm melatonin to the maturation media during the later stages of oocyte maturation (from 22 to 48 h) on the levels of ROS in matured oocytes were measured by DCHF-DA staining at the end of maturation (n = 120; 60 oocytes/treatment group). Additional maturated oocytes were subjected to IVF with the fertilization media supplemented with 5.0 μg/ml tannic acid. Endpoints measured were the number of oocytes penetrated, the number of polyspermic oocytes (of those penetrated), and the number of oocytes penetrated with a MPN (of those penetrated; n = 200; 50 oocytes/treatment group). During embryonic development (n = 400; 100 oocytes/treatment group), the number of embryos cleaved at 48 h after IVF and blastocysts at 144 h after IVF were observed and recorded. A total of 720 matured oocytes (180 oocytes/treatment group) over three replicates were used in this experiment.

### Statistical analysis

Data were analyzed by one-way ANOVA using the PROC GLM procedures of SAS (SAS Institute, Cary, NC, USA). When there was a significant effect, significant differences were determined using the LSMEANS statement and Tukey adjustment for multiple comparisons. The effects included in the initial model were treatment, well and replicate. Well and replicate effects were not significant (P > 0.05) and were deleted from the finals models. Chi-square analysis was used to determine percentages of embryos reaching the different developmental stages for each treatment. In all analyses, P < 0.05 was considered to be significant. Results are expressed as the least-squares mean ± SEM.

## Results

### Experiment 1: determination of optimum concentration of melatonin for embryo production

Oocytes supplemented with 75 nm melatonin had significantly lower (P < 0.05) incidences of polyspermic penetration (13.75 ± 3.11%) compared to all other treatment groups and significantly higher (P < 0.05) MPN formation (66.25 ± 9.29%) compared to oocytes supplemented with 150 nm melatonin (27.50 ± 10.84%; Table 1 ).

**Table 1:** Effects of melatonin supplementation on oocyte fertilization characteristics 12 h after fertilization.

Treatment group †	Oocytes penetrated (%)	Polyspermic oocytes § (%)	Oocytes with MPN § (%)
No melatonin	86.00 ± 8.18	25.58 ± 8.72^a^	47.67 ± 9.34^ac^
75 nm melatonin	80.00 ± 8.18	13.75 ± 3.11^b^	66.25 ± 9.29^ab^
100 nm melatonin	80.00 ± 8.18	30.00 ± 7.14^a^	80.00 ± 4.54^b^
150 nm melatonin	85.00 ± 8.18	22.35 ± 4.33^a^	27.50 ± 10.84^c^

† Treatment groups were the final concentration of melatonin supplemented to the oocyte maturation media in the second phase of maturation.

§ Percentage of the number of oocytes penetrated.

a,bMeans within a column with different superscripts differ significantly (P < 0.05). Differences between columns are not comparable. Data are expressed as Mean ± SEM.

**Figure 1:**
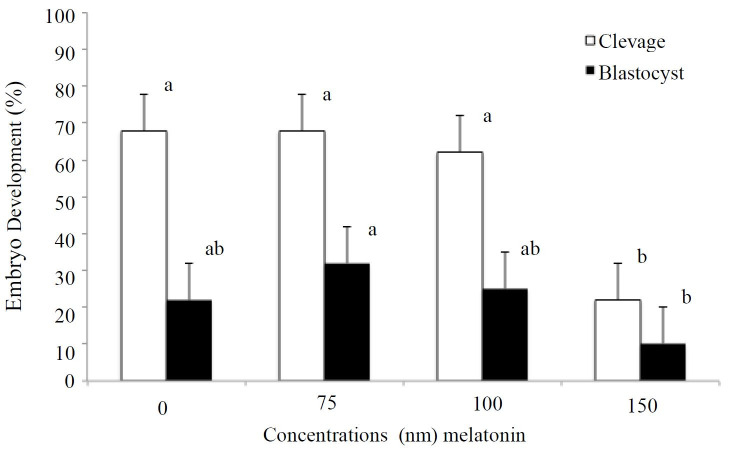
Effects of different melatonin concentrations supplemented in the oocyte maturation media at 22 h on embryo development (n = 400) in experiment 1. Cleavage, observed 48 h after IVF; blastocysts, observed 144 h after IVF. ^a,b^Means with different superscripts differ at least P < 0.05. Differences between cleavage and blastocyst are not comparable. Data expressed as Mean ± SEM.

Embryo development results are shown in Fig. 1 . There were no differences in the percentage of embryos cleaved by 48 h between no melatonin supplementation, 75 nm or 100 nm supplementations. Oocytes supplemented with 150 nm melatonin had a significantly fewer (P < 0.05) percentage of embryos cleaving by 48 h after IVF (22.00 ± 21.37%) compared to all other treatment groups. Oocytes supplemented with 75 nm melatonin had a significantly higher (P < 0.05) percentage of embryos reaching the blastocyst stage of development by 144 h after IVF (32.00 ± 17.32%) compared to oocytes supplemented with 150 nm melatonin (10.00 ± 17.32%).

### Experiment 2: effects of melatonin and tannic acid supplementation on IVF and embryonic development

Supplementation of 75 nm of melatonin was the lowest supplementation level that did not have detrimental effects on sperm penetration, MPN formation, cleavage and blastocyst formation and thus was the supplementation level used in experiment 2.

Oocytes supplemented with 75 nm melatonin significantly decreased (P < 0.05) ROS generation (67.00 ± 3.02%) compared to oocytes with no melatonin supplementation (100 ± 3.02%). There were no significant differences in penetration rates of the oocytes however oocytes fertilized with sperm incubated with tannic acid had a significantly less (P < 0.05) incidence of polyspermic penetration. All treatment groups had significantly greater (P < 0.05) incidence of male pronuclear formation compared to oocytes not supplemented with melatonin and fertilized without tannic acid ( Table 2 ).

**Table 2:** Effects of melatonin and tannic acid supplementation on oocyte fertilization characteristics 12 h after fertilization.

Treatment group †	Oocytes penetrated (%)	Polyspermic oocytes § (%)	Oocytes with MPN § (%)
No melatonin, no tannic acid	84.00 ± 3.50	33.33 ± 2.10^a^	66.67± 5.51^a^
75 nm melatonin, no tannic acid	80.00 ± 5.30	27.50 ± 4.11^a^	82.50 ± 4.37^b^
No melatonin, 5.0 μg/ml tannic acid	90.00 ± 5.58	17.78 ± 4.09^b^	84.44 ± 3.53^b^
75 nm melatonin, 5.0 μg/ml tannic acid	84.00± 2.00	16.67 ± 5.85^b^	85.71 ± 4.35^b^

† Treatment groups were the final concentration of melatonin supplemented to the oocyte maturation media in the second phase of maturation, and the final concentration of tannic acid supplemented to the IVF media during sperm thawing and IVF.

§ Percentage of the number of oocytes penetrated.

a,bMeans within a column with different superscripts differ significantly (P < 0.05). Differences between columns are not comparable. Data are expressed as Mean ± SEM.

**Figure 2:**
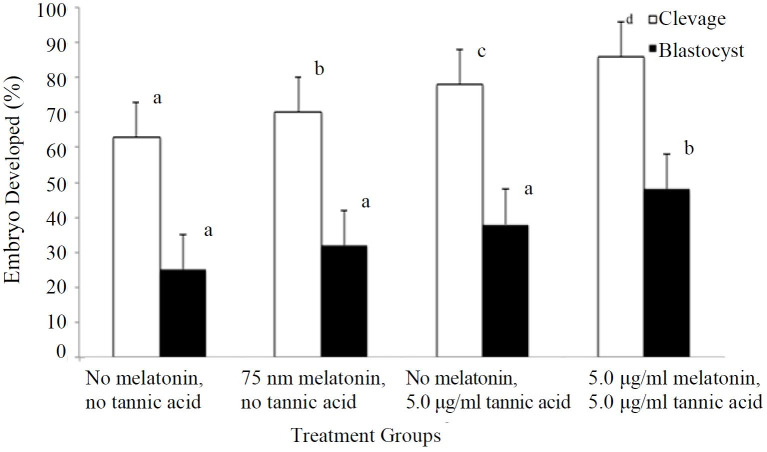
Effects of 75 nm melatonin supplemented in the oocyte maturation media at 22 h and 5.0 μg/ml tannic acid supplemented to the IVF media during sperm thawing and IVF on embryo development (n = 400) in experiment 2. Cleavage, observed 48 h after IVF; blastocysts, observed 144 h after IVF. ^a,b^Means with different superscripts differ at least P < 0.05. Differences between cleavage and blastocyst are not comparable. Data expressed as Mean ± SEM.

There were significant differences (P < 0.05) in cleavage rates by 48 h post-IVF between each of the groups ( Fig. 2 ). Oocytes supplemented with melatonin and fertilized with sperm incubated with tannic acid had a significantly higher (P < 0.05) percentage of blastocyst formation by 144 h post-IVF (48 ± 4.04%) compared all other treatment groups ( Fig. 2 ).

## Discussion

Despite continual advancements, IVF of porcine oocytes continues to be a challenge owing to poor cytoplasmic maturation, high incidences of polyspermic penetration, and elevated levels oxidative stress during maturation, which often leads to higher levels of ROS production ( Abeydeera, 2002 ; Gil *et al*., 2010 ; Dang-Nguyen *et al*., 2011 , Alvarez *et al*., 2015 ). Embryonic development is impacted by the quality of the spermatozoa used in fertilization as well as environmental conditions ( Wang *et al*., 2003 ; Gil *et al*., 2008 ). Pigs typically have a high amount of oxygen tension that is present during *in vitro* maturation, which accumulate ROS inside the oocyte and cause damage to the DNA, which impairs embryo development ( Kitagawa *et al*., 2004 ).

Previous research has indicated that the supplementation of antioxidants such as melatonin may decrease ROS and improve subsequent embryonic development in multiple species ( Cebrian-Serrano *et al*., 2013 ; Salehi *et al*., 2014 ). Additionally, melatonin has been shown to exist in the follicular fluid surrounding pig oocytes ( Shi *et al*., 2009 ). Supplemention 1 nm melatonin during oocyte maturation and embryo culture improved cleavage and blastocyst formation rates *in vitro* ( Shi *et al*., 2009 ). In agreement with those studies, our results indicated that supplementing melatonin during oocyte maturation improved early embryonic development rates.

Melatonin has also been shown to reduce levels of ROS and enhance glutathione production and decrease apoptosis in pig oocytes ( Li *et al*., 2015 ). Our results agree with this study, as we found supplementation of melatonin to decrease the ROS produced during maturation. The level of melatonin appears to impact the success of oocyte maturation, as a previous study reported minimal improvements, however the levels were lower than those used in the current study ( Choe *et al*., 2010 ). Shi *et al*. (2009) reported that the concentrations of melatonin change as the follicle changes size, suggesting an effect of melatonin in oocyte maturation: as the follicle size increased, the levels of melatonin decreased. In addition to alleviating oxidative stress in oocytes, Jang *et al*. (2010) demonstrated that when melatonin was supplemented to boar semen, semen characteristics and developmental quality of *in vitro* derived embryos improved.

Supplementation of 5µg/ml tannic acid to the sperm thawing and fertilization media decreases polyspermic penetration in porcine oocytes ( Tatemoto *et al*., 2006 ). Tannic acid has antihyaluronidase activity and scavenges ROS in boar sperm, which has shown to improve IVF in pigs ( Tokeshi *et al*., 2007 ). Our results were similar, as supplementation of tannic acid to the fertilization media decreases polyspermic penetration regardless if melatonin was supplemented during maturation. Matured oocytes supplemented with 75 nm melatonin and fertilized in media containing of 5 µg/ml tannic acid had significantly higher cleavage rates at 48 h post-IVF and blastocyst formation by 144 h post-IVF than no supplementations or supplementations of only melatonin or tannic acid.

Although our results and Tatemoto’s *et al*. (2006) indicate that tannic acid supplementation during IVF reduces polyspermic penetration in pigs, more studies should be conducted to determine if supplementing tannic acid during oocyte maturation or during the freezing process of semen has the ability to further improve fertilization success and embryonic development. Varying the concentration of tannic acid during IVF could change the success rates ( Li *et al*., 1997 ) so further studies should be conducted to determine if there is species or even boar variability. Melatonin supplementation is currently a popular topic of interest and appears to improve multiple mechanisms in the oocyte, including meiotic maturation ( Park *et al*., 2017 ), embryonic development (Chen *et al.*, 2017), and lipid metabolism ( He *et al*., 2018 ). Despite the promising effects of melatonin supplementation, its specific mechanism(s) of action on the pig oocyte are relatively unknown. More research needs to be done to elucidate how melatonin affects the oocyte and if there is an endogenous role or it is entirely a supplemental effect.

Since the continual production of high quality porcine embryos has enumerable benefits, it would be advantageous for the development of a chemically defined *in vitro* system. Notwithstanding extensive research, polyspermic penetration is still a major issue in IVF for the porcine species, which is a critical component in the process of development. To our knowledge, this is the first study to provide information on the effects of melatonin supplementation to the maturation media with tannic acid supplementation to the fertilization media of pig oocytes. Our results indicate that supplementation with melatonin and tannic acid have beneficial effects on reducing polyspermic penetration and improving early embryonic development. Further work needs to be conducted to determine the specific mechanisms of action of melatonin on the oocyte and its surrounding environment. These data will improve our knowledge of alleviating polyspermy associated with IVF and could be used to develop new methods to modify the IVF conditions to improve the *in vitro* production of pig embryos.
